# The burden of drug resistant tuberculosis in a predominantly nomadic population in Uganda: a mixed methods study

**DOI:** 10.1186/s12879-021-06675-7

**Published:** 2021-09-14

**Authors:** Brenda Nakafeero Simbwa, Achilles Katamba, Elizabeth B. Katana, Eva A. O. Laker, Sandra Nabatanzi, Emmanuel Sendaula, Denis Opio, Jerry Ictho, Peter Lochoro, Charles A. Karamagi, Joan N. Kalyango, William Worodria

**Affiliations:** 1 Clinical Epidemiology Unit, Makerere University College of Health Sciences, Kampala, Uganda; 2 Infectious Disease Institute, Kampala, Uganda; 3 Department of Medicine, Makerere University College of Health Sciences, Kampala, Uganda; 4 Division of Pulmonology, Department of Medicine, Mulago National Referral Hospital, Kampala, Uganda; 5 Department of Pediatrics, Makerere University, Kampala, Uganda; 6 Doctors with Africa, CUAMM, Kampala, Uganda

**Keywords:** Drug resistant Tuberculosis, Nomadic, Uganda, Karamoja, Gene-Xpert, Low prevalence, Alcohol, Drug stock out, Stigma

## Abstract

**Background:**

Emergence of drug resistant tuberculosis (DR-TB) has aggravated the tuberculosis (TB) public health burden worldwide and especially in low income settings. We present findings from a predominantly nomadic population in Karamoja, Uganda with a high-TB burden (3500 new cases annually) and sought to determine the prevalence, patterns, factors associated with DR-TB.

**Methods:**

We used mixed methods of data collection. We enrolled 6890 participants who were treated for tuberculosis in a programmatic setting between January 2015 and April 2018. A cross sectional study and a matched case control study with conditional logistic regression and robust standard errors respectively were used to the determine prevalence and factors associated with DR-TB. The qualitative methods included focus group discussions, in-depth interviews and key informant interviews.

**Results:**

The overall prevalence of DR-TB was 41/6890 (0.6%) with 4/64,197 (0.1%) among the new and 37/2693 (1.4%) among the previously treated TB patients respectively. The drug resistance patterns observed in the region were mainly rifampicin mono resistant (68.3%) and Multi Drug-Resistant Tuberculosis (31.7%). Factors independently associated with DR-TB were previous TB treatment, adjusted odds ratio (aOR) 13.070 (95%CI 1.552–110.135) and drug stock-outs aOR 0.027 (95%CI 0.002–0.364). The nomadic lifestyle, substance use, congested homesteads and poor health worker attitudes were a great challenge to effective treatment of TB.

**Conclusion:**

Despite having the highest national TB incidence, Karamoja still has a low DR-TB prevalence. Previous TB treatment and drug stock outs were associated with DR-TB. Regular supply of anti TB medications and health education may help to stem the burden of TB disease in this nomadic population.

## Background

Worldwide Tuberculosis (TB) is one of the leading infectious diseases with the highest case fatality rate [1]. In Sub Saharan Africa, drug resistance is becoming a great challenge in the fight against TB [2]. Multidrug-resistant tuberculosis (MDR-TB) a global public health problem, is caused by TB bacteria that is resistant to at least isoniazid and rifampicin [3]. DR-TB is transmitted through air from person to person but may sometimes develop when the bacteria becomes resistant to the drugs used to treat TB as a result of poor adherence to or wrong prescription of TB treatment [2]. In many countries, treatment of MDR-TB is more than seven times the treatment of susceptible TB and this may deter effectiveness of TB control programs [4, 5]. This is because MDR-TB involves expensive second-line regimens given the prolonged treatment [6]. However this poses serious financial challenges for families and health systems as a whole, especially in sub-Saharan Africa with economically constrained settings [7]. The World Health Organization (WHO) anti-TB drug resistance surveillance data shows that there’s an increasing number of new and previously treated TB cases in the world that have rifampicin or multidrug-resistant tuberculosis [8]. The latest treatment outcome data shows over half of the Multidrug-resistant/Rifampicin Resistant tuberculosis (MDR/RR-TB) patients who start treatment are successful, mortality rate is at 18%, and those that fail on treatment are 8%. [9] However, treatment success in extensively drug-resistant TB patients is only a third [9]. Extensively drug-resistant TB (XDR-TB) is a rare type of MDR-TB that is resistant to isoniazid and rifampin, plus any fluoroquinolone and at least amikacin, kanamycin, or capreomycin [10]. In 2017, WHO reported about 558,000 new cases of MDR/RR-TB and a Case fatality rate of 40% globally and 8.5% of these cases had extensively drug-resistant TB (XDR-TB). In the same period TB was responsible for 1.3 million HIV-negative and 374,000 HIV-positive deaths globally [11]. This indicates TB is fatal and prevention efforts should be emphasized.

Karamoja, in north-eastern Uganda, is considered by many a “hard to reach” sub-region [12]. It is characterised by cattle rustling, insecurity from armed nomadic tribes, a semi-arid climate and a few economic activities [12] of which the local community has not been involved extensively. For years, infrastructure including roads, healthcare facilities and adequate water had been specially lacking, making the sub-region the least socially and economically developed in Uganda [13]. Karamoja is the region with the highest incidence of TB in Uganda [14] with 3500 new cases identified and treated every year including increasing cases of MDR [15]. Unfortunately, this is due to people who stop their treatment early. An estimated 40% of the TB patients fail to complete their course of treatment and abandon health care facilities [14, 15]. The cost of treating a patient for drug-susceptible TB is estimated to be 258 US dollars in Uganda, however treating an MDR patient can cost more than 1200 US dollars [16] which is beyond the reach of the average Ugandan [17]. Some of the specific risk factors for TB in nomadic populations include poor living conditions (small and crowded group of huts within one fence, the Manyattas), Poor adherence due to the long distances travelled while raring cattle, TB knowledge gaps [18], higher perceived stigma [19], working conditions associated with high risk of TB transmission like working in the mines with crowded spaces and poor ventilation [20]; and other factors such as diseases that damage immunity, malnutrition, smoking, and alcohol abuse [15, 21]. Although a national survey on drug resistance prevalence, patterns and associated has been done [22], among nomadic populations in Uganda, the patterns and associated factors of DR TB have not been documented. There still exist substantial knowledge gaps on anti-TB-drug resistance that ought to be addressed. It’s therefore important that the burden and risk factors of DR-TB in nomadic populations are determined in order to inform policy makers of status of this community and the existing gaps in programming for their DR TB prevention needs. This study aimed to determine the prevalence, patterns and factors associated with and factors influencing DR-TB in a predominantly nomadic population in Uganda.

## Materials and methods

### Study design

A baseline cross-sectional study was carried out in Matany hospital to determine the prevalence of drug resistant tuberculosis in Karamoja region. A case control study and qualitative study were then carried out to determine the factors associated with drug resistant tuberculosis in Karamoja region among TB patients in the period of January 2015 to January 2018.

### Study setting

Karamoja region has seven districts with 37 subcounties. The study was conducted at St. Kizito Hospital Matany in Napak district. Matany Hospital is the only DR-TB treatment initiation site in Karamoja region. The Hospital has been Gene-Xpert (gold standard) for diagnosing TB as well as rifampicin resistance since 2015. This has made Matany Hospital a diagnostic and centre for MDR-TB. The hospital also treats all DR-TB patients from the region including adults and children. Patients diagnosed with rifampicin resistant TB on the Xpert® MTB/RIF assay are subjected to additional investigations including sputum culture, drug susceptibility testing, chest X-rays, HIV tests, thyroid function tests and blood chemistry tests. Patients are then started on the national standardized MDR-TB treatment regimen while awaiting sputum culture and drug susceptibility testing results. The drug susceptibility test results inform on the pattern of resistance either resistance to rifampicin +/− isoniazid (MDR) or resistant to fluoroquinolone (XDR).

### Data collection and management

#### Cross sectional study

Data to determine the prevalence and patterns of DR-TB was extracted from the District Health Information System (DHIS2) online tool. Data of all TB patients between January 2015 and April 2018 was extracted and analysed by the PI.

#### Case control study

A matched case–control study among DR-TB patients was conducted retrospectively. Cases were matched with controls by Location (Place of residence) at Sub-county level. We matched to control for Sex, Age and area of residents.

We defined a case as a TB patient with sputum culture positive for *Mycobacterium tuberculosis*, resistant to at least isoniazid and rifampicin and a control as a sputum smear-positive drug-susceptible TB patient.

All cases at Matany hospital initiation site were enrolled in the study since the numbers of cases were few (41). Four controls were randomly enrolled per case identified from the sampling frame containing eligible controls. Simple random sampling procedures using computer generated random numbers were used to select controls who presented around same time of 2 months as the case identified. In the event that random number was repeated or control died or migrated, the immediate next person in the TB register was considered.

For each participant we collected the following information; social demographic characteristics, HIV status, nutrition status at diagnosis, history of TB treatment, disease classification and drug stock outs in facilities where patient was getting treatment using the health facility TB registers and anti TB drug stock cards used from January 2015 to April 2019.

The research assistants extracted data from the patient files and TB registers using a pre designed questionnaire. This was used to collect the quantitative data. Prior to the beginning of data collection, the questionnaire was pretested in a sample of 30 records in OPD and all inconsistencies noted in the line of data collection were corrected. Research assistants were trained on the methods of data collection to extract the data from the databases.

Triangulation of the information from the different sources was undertaken to ensure consistency.

#### Qualitative study

We conducted a qualitative study with the aim of assessing the perspectives, opinions, social and cultural factors influencing and associated with drug resistant tuberculosis among Tuberculosis patients in Karamoja region. The qualitative interview guides were specifically developed for each specific interview but was reviewed and approved by relevant research ethics committees, and pre-tested in the Wakiso district to assess its suitability.

Ten Key informant interviews were carried with Health workers in the seven districts to assess heath related factors influencing drug resistant tuberculosis. The participants included Medical officers in charge of the TB wards, District Tuberculosis and Leprosy Supervisors, Health Inspectors, Nurses, Clinical Officers, data managers and District Health Officers. These were carried out by the PI in English. They were all recorded and notes were taken during the interviews. These were 10–15 min long.

#### In-depth interviews

Ten in-depth interviews were carried out with patients with DR-TB to determine social–cultural factors influencing and associated to drug resistance. These were carried out in both English and the local language. An in-depth interview tool guide was used to guide the discussion and the discussion points in the guide were those that could help to describe the socio-cultural factors influencing Drug resistance among patients with TB. The interviewers were required to be thoroughly familiar with the in-depth interview tool as it allowed the moderator to be more engaged during the discussion and to rephrase questions that were unclear to participants, or to spontaneously think of follow-up questions and probes. The interview took 20–30 min.

#### Focus group discussions

3 Focus Group Discussions (FGD) were carried out to collect data on social–cultural factors influencing and associated to drug resistance. We used Purposive sampling to include participants in a FGD and a homogeneous groups that brings together people of similar backgrounds and experiences was considered. Two skilled moderators conducted the FGDs; one person acted as the moderator of the discussion and the other recording using a voice recorder. An FGD guide was used to guide the discussion and the discussion points in the guide were those that could help to describe the socio-cultural factors influencing Drug resistance among TB patients. The FGD moderators were required to be thoroughly familiar with the FGD guide as it allowed the moderator to be more engaged during the discussion and to rephrase questions that were unclear to participants, or to spontaneously think of follow-up questions and probes [23]. The discussions was audio taped, and notes taken during each session lasting between 45 and 60 min. A total of 10 participants were allowed for each FGD session and the FGDs conducted until there was data saturation therefore preliminary was data reviewed and analysis was done in conjunction with data collection.

### Study variables

#### Dependent variable

The Primary outcome in this study was Drug resistance to anti-TB drugs. It was measured as a categorical variable.

#### Independent variables

##### Patient socio-demographic factors

Age, sex, marital status of the cases and control. These were measured as categorical variables. Residence of the cases and controls; was measured as a continuous variable.

##### Clinical factors

Type of patient, HIV status, Nutrition status, Disease classification of the subjects and these were measured as categorical variables.

##### Drug related factors

Drug stock out; this was measured as a categorical variable to determine whether a health facility had any TB drug stock outs in the period between January 2015 and January 2018.

##### Substance use

Alcohol consumption, Herbal medicines and smoking status of the subjects were measured as categorical variables.

The factors associated with Drug resistant Tuberculosis are showed in Fig. 2 as well as the secondary outcomes.

## Data management

Interviews were recorded, transcribed and analysed using open code. Quantitative data was double entered, cleaned, and edited in a statistical software EpiData version 3.1 and thereafter exported to STATA version 13.0. Adjustment was made for the effect of clustering in the data and all statistical analysis performed using STATA version 13.0. Discrepancies were checked against the raw data.

All methods were performed in accordance with the relevant guidelines and regulations under the declaration of Helsinki.

### Data analysis

We performed descriptive statistical analysis including frequency counts and percentages for all the participants’ characteristics. To identify the factors associated with drug resistant Tuberculosis, we performed conditional logistic regression analysis with a significance level of 0.05. The McNemar’s test (chi-square test for matched-pair data) was used to compare if the cases are significantly different from the controls. We conducted both unadjusted and adjusted conditional logistic regression with the adjusted model only including factors that are significant in the unadjusted model. The goodness of fit for the adjusted model was tested through the link-test goodness of fit. For qualitative data, we undertook thematic-content analysis for the qualitative data using Open code version 4.2.1 to generate themes from the interviews and followed by interpretation of the data generated.

### Study participants flow chart

**Fig. 1:**
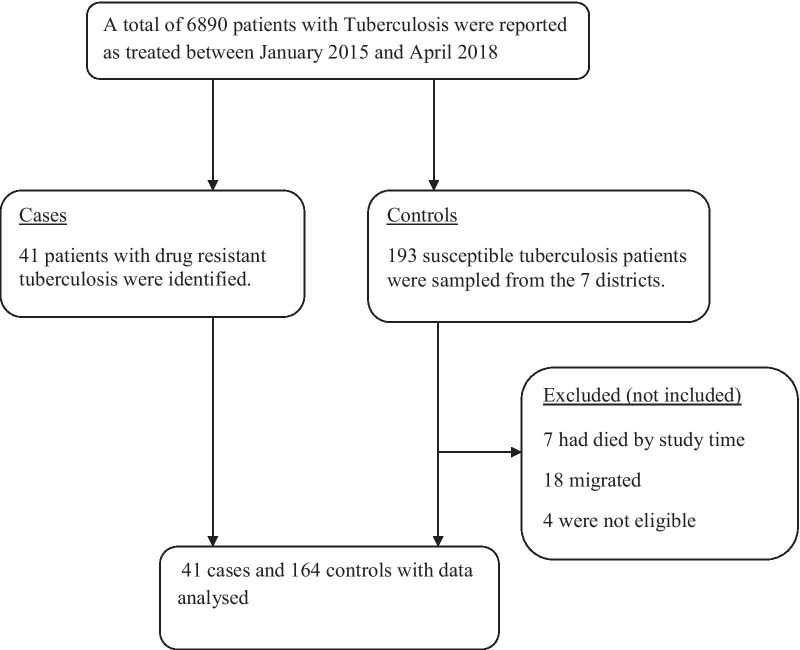
Patient flow chart during the study in Karamoja region, 2018

**Fig. 2:**
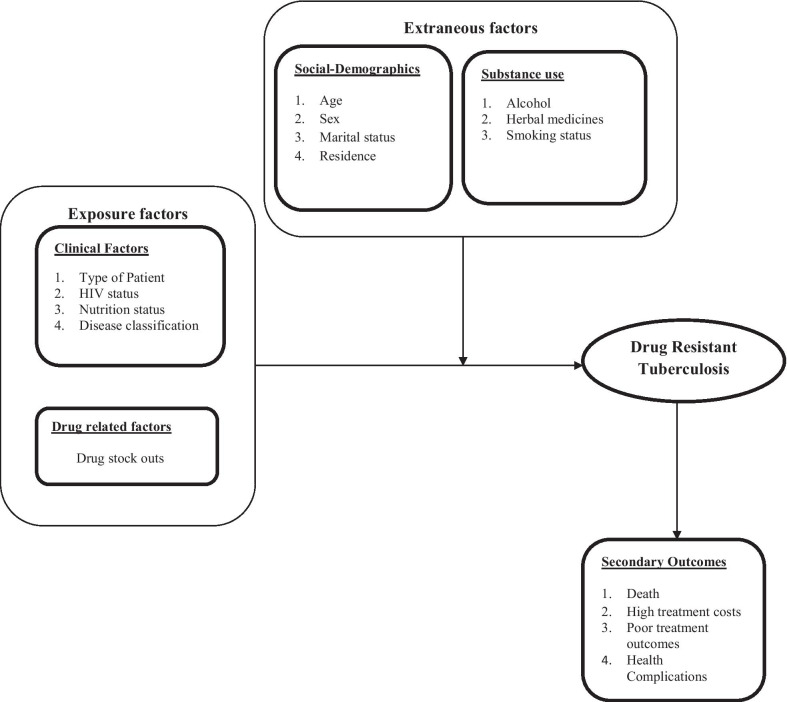
Conceptual framework showing the possible factors associated with drug resistance

A total of 6890 people were reported to have received treatment for Tuberculosis in the period between January 2015 and April 2018. Of these 41 patients were found to have drug resistant tuberculosis. All the 41 patients and 164 controls were included in our study as showed in Fig. 1

The analysis was completed on 41 cases and 164 controls.

## Results

The prevalence of DR-TB among patients with TB in Karamoja region in the period between January 2015 and April 2018 was 0.6%. Results of DR-TB are summarised in Table 1.

**Table 1:** Prevalence of drug resistant tuberculosis among patients with tuberculosis in Karamoja region

Variable	Frequency (n)	Percentage (%)
Overall		
n = 6890	41	0.60
According to patient type		
New (n = 4197)	4	0.1
Previous TB treatment (n = 2693)	37	1.4
Age		
≤ 9 years (n = 756)	1	0.1
10–19 years (n = 1268)	5	0.4
20–59 years (n = 4134)	29	0.7
≥ 60 years (732)	6	0.8
Sex		
Male (n = 4479)	19	0.4
Females (n = 2411)	22	0.9
District of residence		
Abim (n = 584)	2	0.03
Amudat (n = 150)	1	0.07
Kaabong (n = 823)	4	0.05
Kotido (n = 721)	6	0.08
Moroto (n = 1523)	2	0.01
Nakapiripirit (n = 1161)	16	0.14
Napak (n = 1928)	10	0.05

Among the TB patients s with drug resistance in region, majority 28 (68.3%) had rifampicin mono-resistance while 13 (31.7%) had MDR-TB in Karamoja as summarised in Table 2.

**Table 2:** Patterns of drug resistance among patients with tuberculosis in Karamoja region

Characteristics	RR n (%)	MDR n (%)
Overall		
Frequency (n)	28 (68.3)	13 (31.7)
Age		
≤ 19 years	5 (17.9)	1(7.7)
20–59 years	21 (75.0)	8 (61.5)
≥ 60 years	2 (7.1)	4 (0.3)
Sex		
Males	13 (46.4)	6 (46.2)
Females	15 (53.6)	7 (53.8)
Districts		
Abim	0 (0.0)	2 (15.4)
Amudat	1 (3.6)	0 (0.0)
Kaabong	2 (7.1)	2 (15.4)
Kotido	4 (14.3)	2 (15.4)
Moroto	1 (3.6)	1 (7.7)
Nakapiripirit	13 (46.4)	3 (23.1)
Napak	7 (25)	3 (23.1)

The median age of participants was 37.5 years (interquartile range (IQR) 25 ranging from 6 to 80 years with more females (53.7%) having TB. The greatest proportion of TB patients (39%) was from Nakapiripirit district and was among married participants. A large percentage of the participants had a previous history of TB and was malnourished. Substance use was high among the participants and a small proportion (2.4%) had HIV. Most of the participants received services from a health centre that had at least one TB drug stock-out as shown in Table 3.

**Table 3:** Characteristics of demographic, social economic, and health services-related factors for 41 cases and 164 controls in Karamoja region

Characteristics	Frequency (n)	Percentage (%)	P value
	Cases (N = 41)	Controls (N = 164)	
Marital status			
Married n (%)	26 (63.4)	126 (76.8)	0.034
Not married n (%)	15 (36.6)	38 (23.2)	
Personal health-related factors			
Category of patient			
New n (%)	4 (9.8)	156 (95.1)	< 0.001
Previous TB treatment n (%)	37 (90.2)	8 (4.9)	
HIV status			
Negative n (%)	39 (95.1)	156 (95.1)	1.000
Positive n (%)	2 (4.9)	8 (4.9)	
Malnutrition			
Yes n (%)	24 (58.5)	82 (50.0)	0.363
No n (%)	17 (41.5)	82 (50.0)	
Disease classification			
PTB n (%)	40 (97.6)	120 (73.2)	0.011
EPTB n (%)	1 (2.4)	44 (26.8)	
Substance use			
Yes n (%)	40 (97.6)	95 (57.9)	0.001
No n (%)	1 (2.4)	69 (42.1)	
Health services factors			
Drug stock out			< 0.001
Yes n (%)	36 (87.8)	36 (22.0)	
No n (%)	5 (12.2)	128 (78.0)	

Marital Status (OR 3.16), disease classification (OR 17.09), category of TB patient (OR 7.199) and substance use (OR 1.798) and were found significantly associated with DR-TB. Among the health services factors, only drug stock out (OR 0.023) was found significant as shown in Table 4.

**Table 4:** Bivariate analysis between independent factors and drug resistant tuberculosis

Variable	DR-TB		OR	95% CI	P
Socio-demographic factors	Cases n (%)	Controls n (%)			
Marital status					
Married	26 (63.4)	126 (76.8)	1		
Not married	15 (36.6)	38 (23.2)	3.16	1.09–9.14	**0.034**
Clinical factors					
Category of patient					
New	4 (9.8)	156 (95.1)	1		
Previous TB treatment	37(90.2)	8 (4.9)	7.20	3.47–14.91	** < 0.001**
HIV status					
Negative	39 (95.1)	156 (95.1)	1		
Positive	2 (4.9)	8 (4.9)	1	0.26–6.22	1
Malnutrition					
Yes	24 (58.5)	82 (50.0)	1		
No	17 (41.5)	82 (50.0)	1.44	0.66–3.16	0.363
Substance use					
No	1 (2.4)	31 (18.9)	1		
Yes	28 (68.3)	126 (76.8)	38.23	4.34–336.36	**0.001**
Health services factors					
Drug stock out					
No	5 (12.2)	128 (78.0)	1		
Yes	36 (87.8)	36 (22.0)	43.97	10.43–185.37	** < 0.001**

Bold is for variables with statisttically significant P values

In the CLR model, the aOR of two risk factors were found statistically significantly associated with DR-TB namely, Type of patient and Drug stock out. The aOR of history of previous TB treatment was 5.51 (95%CI 1.74–17.44) compared to those with no history of TB treatment. Thus the risk of DR-TB increased 5.5 times among the respondents who had taken TB treatment in the past than the respondents whose did not. Among the TB patients, the risk was 70% lower among people who were getting treatment from facilities with no TB drug stock out compared to people who were getting treatment from facilities with TB drug stock outs.Substance use was found to confound Drug stock as shown in Table 5.

**Table 5:** Multivariate analysis of independent factors and Drug resistant Tuberculosis in Karamoja

Variable	OR	95% CI	P	AOR	95%CI	P
Marital status						
Married	1			1		
Not married	3.16	1.09–9.14	**0.034**	1.84	0.30–11.25	0.510
Category of patient						
New	1			1		
Previous TB treatment	7.20	3.47–14.91	** < 0.001**	5.54	1.74–17.44	**0.004**
Drug stock out						
No	1	1		1		
Yes	43.97	10.43.185.37	** < 0.001**	20.1	3.62–112.57	**0.001**
Confounders substance use						
No	1			1		
Yes	38.23	4.34–336.36	**0.001**	3.35	0.44–25.7	0.0.0.246

Bold is for variables with statisttically significant P values

## Qualitative findings

### The key themes that emerged from the qualitative analysis

The key issues that emerged in relation to Drug Resistant Tuberculosis in Karamoja District are as outlined below.

#### Drug resistance still a big problem

Drug resistance is a big issue among the nomadic in Karamoja and the numbers reported are much less than the actual numbers. This is because of the long distances travelled to access health services, poor retention, patients with TB who do not report to health facilities at all and their nomadic life style.

“…most of the patients walk a long distance to the health centres to get medication. They stay in very remote areas with very bad roads especially in rainy seasons even rivers follow and block them.” *Key informant from Nakapiripirit district*.

“….Karamojongs are pastoralists and yet the weather here is not very favourable so they usually travel in groups and make kraals where there’s food for the cattle sometimes it is even in Kenya, we have failed to find such people” *Key informant from Napak district*.

#### Congested homesteads

Poor housing conditions in Karamoja have increased the exposure of disease to other people.

The Karamojongs stay in enclosed homesteads with many people sharing poorly ventilated huts.

“…At my home we sleep 15 girls in my hut and in the Manyatta we have very many families. All of us young girls have to sleep in one hut. Only those who are going to get married are given their own huts” *A participant during FGD with DR-TB patients in Napak district*.

#### Retention in care for susceptible TB patients

Retention in care for susceptible TB patients is very poor. The rate of lost to follow up and treatment failure is very high in the region. During the dry season the nomads migrate to look for greener pastures for their animals. Most patients with TB also move without completing the treatment and rarely visit health facilities where they have migrated to.

“…some of the problems we are facing with TB treatment here is lost to follow up most of our people don’t have mobile phones so it is very hard to contact them when they don’t come for appointments” *Key informant from Kotido district*.

“…The Pastoralists go to the kraals for months and return only if they are very sick or the dry season is done. Even then we do not know the exact time and period they go and yet they don’t inform us.” *Key informant from Moroto district*.

#### Direct observed treatment (DOTs) program is not well implemented

All the patients with TB are eventually enrolled on community based DOTs program and yet they have no treatment supporters and the rate of lost to follow up is high.

“…here we use the community based DOT because we cannot afford Facility based but even the treatment supporters are not doing their jobs. Some of them can’t remind the person to take his drugs when they are away in the kraals also wives can’t force their husbands to adhere to drugs if they refuse to.” *A participant during FGD with Health workers in Karamoja*.

The DOTs program is also challenged by stigma as awareness is increasing people instead want to stay away from the patients so adherence is not closely monitored

“…the other problem faced by TB patients is isolation and stigma. One chairman told the whole village that a patient had Ebola so they chased him from the village because they didn’t want to be infected too.” *A participant during FGD with Health workers in Karamoja*.

#### Too much substance use

Many of the adults take alcohol in their free time and community hours in the evenings while the pastoralists smoke a lot to keep warm. Also many believe you have you use traditional herbs to get well.

“…it is very hard to stop drinking especially when on medication. But when I go home a drink a little and take the medicine in the morning.” *A participant during FGD with DR-TB patients in Napak district*.

#### Poor adherence to TB medication

There is very poor adherence especially when patients leave hospital to return home and when they go to the kraals. The long regimens and associated side effects make it very hard to finish the medication.

“…in the kraals we sometimes sleep under one cow to protect them. We carry very little clothing. Sometimes the medicine gets lost and you can’t find it.” *A participant during FGD with DR-TB patients in Napak district*.

“The tablets are very many and for a long time. The problem is that you have to keep going to hospital to pick every month yet sometimes we have travelled far from our homes. Also the medication makes me weak and you can’t work yet everyone depends on me” *A participant during FGD with DR-TB patients in Napak district*.

#### Attitudes in the health workers

Some health workers avoid interacting with TB patients for fear of being infected. There is a gap in managing patients with TB.

“Health workers here are not empowered to manage TB patients, they fear to treat them so they isolate the patients especially the recurrent ones.” *Key informant from Moroto district*.

#### Lack of equipment and drug stock out

Most of the health facilities have had more than one episode of drug stock out in a year. The protective gears and Gene-Xpert cartridges can sometimes run out of stock for a long period of time.

“…sometimes the nearby hospital doesn’t have the medicine that it is finished so we have to wait until they bring more. If you feel so sick you take some of our traditional herbs and they work” *A participant during FGD with DR-TB patients in Napak district*.

“…One of the problems we have is that NMS is not consistent with drug supplies so when we run out of anti TB drugs we borrow from nearby Health facilities however sometimes they also don’t have some drugs so we send patients to other facilities, but some of course don’t go and am sure these are the people who get resistance” *Key informant from Nakapiripirit district*.

“…We have equipment but they are not been serviced in a long time even after we have written emails and sent complaints. Sometimes the gene Xpert machine has no cassettes and it takes very long to get them. This decreases case detection”. *A participant during FGD with Health workers in Karamoja*.

## Discussion

The study found a low prevalence of DR-TB among new and previously treated TB patients respectively in Karamoja region. WHO classifies settings with a DR-TB prevalence of less than 3% among new patients as having a low DR-TB burden [11]. These findings were consistent with previous findings of an anti-TB drug resistance survey in Uganda [22]. The low burden observed may be attributed to the low access to health care and movements in the region due to the nomadic life styles.

Nakapiripirit district had the highest number of DR-TB patients (0.14% of its TB patients reported in this period) followed by Napak and Kaabong districts.

These findings are however surprising because Karamoja is a region with the highest TB incidence in Uganda and only 40% of TB patients complete their treatment, majority abandon health care facilities due to their nomadic lifestyle [15]. The region only has two rapid molecular diagnostic test centres and no centres that can perform mycobacteriology cultures this could be a reason for under detection of DR-TB cases. From our study over 90% of the DR-TB patients were previously treated susceptible TB (These include participants with Treatment relapse, Lost to follow up and Treatment failure) therefore prevalence of DR-TB maybe higher than observed.

DR-TB patients that were identified as MDR-TB were 31.7% and an additional 68.3% patients were found to have resistance to rifampicin placing these patients just one step away from developing MDR-TB these results are in line with the Global TB report 2017 that showed that there are more RR patients than MDR-TB patients [11].

Previous history of TB treatment was significantly associated with DR-TB. Patients who had previous history of treatment for TB were 5.51 times at higher risk of developing MDR-TB than patients who did not have a history of previous treatment for TB this is also consistent with findings from studies in Ethiopia [22, 24, 25] who reported that most of the people that were diagnosed with DR-TB had a history of having received treatment for TB.

Similar to previous studies, HIV status had no significant association with DR-TB [22, 25]. However this is contrary to the study in Ethiopia which found that patients who had HIV infection had three times higher risk than those who had no HIV infection to develop DR-TB [24]. They noted that this association had a marginal statistical significance showing that HIV infection is not a strong predictor of DR-TB infection in TB patients. Karamoja is one of the regions in the country with the lowest HIV prevalence of 3.4% [26].

In this study substance use was found to be significantly associated with DR-TB. Substance use increased the risk of developing DR-TB by 30%. However, majority of the DR-TB patients (70%, n = 29) were found to consume alcohol. Alcohol consumption is also one of the risk factors for the development of DR-TB. It might be associated with its substantial role in failure rate among new TB cases. Hence, it increases the rate of DR-TB cases. In other studies also alcohol consumption was frequently reported as one of the risk factors for DR-TB in Ethiopia [25].

The nutrition status of TB patients was not significantly associated with DR-TB this may result from the fact that most of the participants were malnourished by the time they were diagnosed. In addition Karamoja region is still battling with food security leaving most families with poor nutrition statuses. These resulted in no difference between the cases and controls.

### Health services factors

Having one or more TB Drug stock outs in health facilities treating susceptible TB was significantly associated with risk of developing DR-TB and this has been noted as one of the factors contributing to poor outcomes and risk for development of DR-TB especially in Health facilities in rural areas in Uganda [27, 28].

### Socio-cultural factors

Evidence from the qualitative method in this study showed that DR-TB is a stigmatized disease in Karamoja. However, the incidence is only increasing due to nomadic movements, poor adherence, poor living conditions where families live in poorly ventilated congested houses in a very close community (Manyattas) with very little economic activity, poor infrastructure, excessive alcohol consumption, several drug stock outs in the health facilities and poor attitudes of health workers.

## Limitations

The study only characterized patients diagnosed through the National Tuberculosis and Leprosy Program supervised health facilities and does not account for drug resistance patterns among patients that did not have access to the health facilities. We do not data about the size and characteristics of these patients.

The study used patients’ self-reports on some variables like substance use which could have caused information bias towards the null.

There could also have been selection bias in this study, as those who had died and those that had migrated and could not be accessed were eliminated. It is possible that these are the patients who had developed Drug resistant TB. However, the impact of this may not have been big because other participants were identified and included in the study.

Health facilities with missing data on anti TB drug stock outs were left out of the study for objective two. It is possible that these facilities had more information on anti-TB drug stock outs that could significantly change our results.

Due to the small numbers of patients with Drug resistant TB the factors discussed as associated with high TB burden may be narrow to explain the MDR-TB patterns.

## Conclusion and recommendations

The national TB program should improve provision of TB drug supplies and expedite the process of decentralization of TB treatment initiation services to lower health facilities. The poor retention due to nomadic lifestyle is high and needs further investigation on when and where the pastoralists migrate to.

## Data Availability

All data generated or analysed during this study are included in this published article. The secondary data used in the study was accessed from the National TB registers in the facility, The anti TB drug stock cards in the Health facilities and from the District Health Information System 2 (DHIS2) with permission from Ministry of health. DHIS2 is a web-based information system used by all health facilities country wide as a health management information system (HMIS).

## Appendix

Conceptual framework (see Fig. 2).

## Abbreviations

AIDS: Acquired Immune Deficiency Syndrome
CBTBC: Community-based tuberculosis (TB) Care
CEU: Clinical Epidemiology Unit
CI: Confidence interval
DOT: Directly Observed Therapy
DR-TB: Drug resistant tuberculosis
DST: Drug susceptibility test
DTLS: District Tuberculosis and Leprosy Supervisor
FGD: Focus Group Discussion
HIV: Human Immuno-Deficiency Virus
IQR: Interquartile range
MDRTB: Multidrug resistant tuberculosis
MOH: Ministry of Health
MSF: Medecins Sans Frontieres International
NTLP: National Tuberculosis and Leprosy Programme
SOMREC: School of Medicine Research Ethics Committee
TB: Tuberculosis
UNCST: Uganda National Council of Science and Technology
WHO: World Health Organization
